# Entomological Investigation and Detection of Dengue Virus Type 1 in *Aedes (Stegomyia) albopictus* (Skuse) During the 2018–2020 Outbreak in Zhejiang Province, China

**DOI:** 10.3389/fcimb.2022.834766

**Published:** 2022-07-01

**Authors:** Qinmei Liu, Jinna Wang, Juan Hou, Yuyan Wu, Hengduan Zhang, Dan Xing, Jian Gao, Chunxiao Li, Xiaoxia Guo, Yuting Jiang, Zhenyu Gong, Tongyan Zhao

**Affiliations:** ^1^State Key Laboratory of Pathogen and Biosecurity, Beijing Key Laboratory of Vector-Borne and Natural Focus Infectious Diseases, Institute of Microbiology and Epidemiology, Beijing, China; ^2^Department of Infectious Diseases Control and Prevention, Zhejiang Provincial Center for Disease Control and Prevention, Hangzhou, China

**Keywords:** dengue virus type 1, *Aedes albopictus*, complete genome, entomological investigation, species composition

## Abstract

Mosquito-borne diseases are still threats to public health in the Zhejiang province of China. Surveillance of mosquitoes and the mosquito-borne pathogen is a vital approach for early warning, prevention, and control of the infectious disease. In this study, from 2018 to 2020, a total of 141607 female mosquitoes were caught by means of the light trap method. The main species were *Culex pipiens quinquefasciatus/pallens* (41.32%), *Culex tritaeniorhynchus* (47.6%), *Aedes albopictus* (2.5%), *Anopheles sinensis* (5.87%), *Armigeres subalbatus* (2.64%) and other mosquito species (0.07%). *Cx. pipiens s.l.* were the dominant species in two urban habitats and rural residential areas while *Cx. tritaeniorhynchus* was the main dominant species in the rural livestock sheds. In terms of seasonal fluctuation, Cx. pipiens s.l fluctuated at a high level from May to October. The peaks of Cx. tritaeniorhynchus, An. sinensis and Ar. subalbatus were in July. In addition, a total of 693 *Ae. albopictus* were collected with Biogents Mosquitaire CO_2_ traps in emergency surveillance of dengue fever (DF) and screened for dengue virus infection. There were three circumstances of collection: The first: the sampling time before mosquito control during the local outbreak of DF in Lucheng of Wenzhou, 2019; The second circumstance: the sampling time after mosquito control during the local outbreak of DF of other cities in 2018-2019; The third circumstance: past DF epidemic areas the sampling time before mosquito control during the local outbreak of DF in Lucheng, Wenzhou, Zhejiang, 2019. The pools formed by mosquitoes collected in these three circumstances were 3 (6.1%), 35 (71.5%), and 11 (22.4%) respectively. Of the 49 pools tested, only one in the first circumstance was positive. The full-length dengue virus sequence of ZJWZ/2019 was obtained by sequencing and uploaded to the NCBI as number OK448162. Full-length nucleotide and amino acid homology analyses showed that ZJWZ2019 and Wenzhou DF serum isolates ZJWZ-62/2019 (MW582816) and ZJWZ-18/2019 (MW582815) had the highest homology. The analysis of full genome and E gene phylogenetic trees showed that ZJWZ2019 belonged to serotype 1, genotype I, lineage II, which was evolutionarily related to OK159963/Cambodia/2019. It implies that ZJWZ2019 originated in Cambodia. This study showed the species composition, seasonal dynamics of mosquitoes in different habitats in Zhejiang province and confirmed the role of *Ae. albopictus* in the transmission cycle of in outbreak of DF in the Lucheng district of Wenzhou in 2019, suggesting the importance of monitoring of vector *Aedes* infected dengue virus in the prevention and control of DF.

## Introduction

Mosquitoes are important arthropods and transmit a variety of diseases including malaria, dengue fever (DF), zika, chikungunya, and Japanese B encephalitis (JE) (WHO, 2020). As climate warming and international travel lead to the outbreak and expansion area of emerging and re-emerging mosquito-borne diseases (Huang et al., 2019), which indicate that mosquito surveillance has become increasingly important. Zhejiang province is located on the southeast coast of China, which has a typical subtropical climate and complex terrain, including plains, hills, and mountains, forming an ideal condition for the habitat of mosquitoes. According to historical and current records of infectious disease reporting, the local public health problems transmitted by mosquitoes include JE (Wang et al., 2009), filariasis (Itoh et al., 2007), malaria (Chen et al., 2015), and DF (Sun et al., 2011). The main vectors are *Culex*, *Anopheles*, and *Aedes*. There are many factors affecting mosquito population dynamics and species distribution, including climate conditions, urbanization, and the local ecological environment (Li et al., 2020). Mosquito population diversity and seasonal fluctuations could in turn affect the risk of mosquito-borne diseases (Mwangangi et al., 2012). Therefore, studies of mosquito species distribution, diversity, and seasonal dynamics could help to formulate and implement better prevention and control strategies for mosquito-borne diseases (Ladeau et al., 2013).

In recent years, DF has gradually become the main mosquito-borne disease in Zhejiang province, with the elimination of filariasis (Itoh et al., 2007) and malaria in China (WHO, 2021), and the reduced incidence rate of JE related to vaccination (WHO, 2019). DF is a mosquito-borne viral disease caused by four dengue virus serotypes. On the first infection, most people have relatively minor symptoms. For many people a second infection is similar to the first but for some people, the second infection can cause severe dengue, and it could be fatal (WHO, 2021). The first outbreak of DF occurred in Ningbo city in northern Zhejiang in 2004 and then in Yiwu city in the inland part of Zhejiang in 2009 (Yan et al., 2010). From 2015 to 2019, 86 of the 90 districts (counties) in Zhejiang province reported cases of DF, of which 44 districts (counties) had indigenous epidemics of DF, and the situation of DF prevention and control was very serious (Wang et al., 2020). Four serotypes of dengue virus have been detected and isolated during outbreaks in Zhejiang province, and dengue virus type 1 (DENV-1) was observed and confirmed in Ningbo city in 2004, DENV-3 in Yiwu city in 2009, and DENV-1 in Wenzhou city and DENV-2 in Shaoxin city in 2009 (Xu et al., 2007; Yang et al., 2009; Sun et al., 2011; Wang et al., 2015; Ren et al., 2018; Yan et al., 2018; Yi et al., 2019; Yu et al., 2019; Lin et al., 2020).

Isolation of dengue virus from patient serum and detecting the virus carried by vector mosquitoes could better grasp the transmission dynamics of DF in many aspects. It is not easy to isolate and detect the virus in vector mosquitoes due to the low infection rate among field vector populations and the emerging response to vector control through space spraying (Balingit et al., 2020; Kosoltanapiwat et al., 2020). Detection of dengue virus in samples of vector *Aedes* mosquitoes collected in the field is useful for the determination of the local transmission cycle and outbreak area (Aranda et al., 2018; Eiras et al., 2018; Kobayashi et al., 2018; Medeiros et al., 2018; Yu et al., 2019; Ferreira-de-Lima et al., 2020; Kosoltanapiwat et al., 2020; Monteiro et al., 2020; Nonyong et al., 2021; Pérez-Pérez et al., 2021; Stephenson et al., 2021); and also can indicate that the serotype and genotype of the virus in mosquito vectors, the distribution area, and the occurrence of vertical transmission in the field. Natural vertical transmission was the observation of larvae (Du et al., 2021), pupae (Martins et al., 2012), and adult males in infected specimens collected in the field; infected larvae, males, and females raised in the laboratory from eggs collected in the field were also considered (Ferreira-De-Lima and Lima-Camara, 2018). Furthermore, it is vital to reveal the trend of dengue epidemics based on phylogenetic analysis and mutations in the gene sequences of viruses (Sittivicharpinyo et al., 2017; Sabat et al., 2019; Punpapong et al., 2020).

At present, vector control is an important part of public health interventions to reduce the spread of mosquito-borne diseases. And the surveillance of mosquitoes and mosquito-borne pathogens is a significant way of early warning, prevention, and control of infectious diseases (Fang et al., 2021). The purpose of this study is to describe the diversity, distribution, and seasonal dynamics of mosquitoes in different habitats in seven prefectures of Zhejiang Province from April to November in 2018-2020, as well as the potential infection and vector role of *Ae. albopictus* during the outbreak of DF.

## Materials and Methods

### Ethics Statement

No specific permits were required for the described field studies: a. No specific permissions were required for these locations/activities, b. These locations were not privately-owned or protected, c. The field studies did not involve endangered or protected species.

### Entomological Investigation

The light trap method, recommended by the Chinese Center for Disease Control and Prevention (China CDC) (National Health Commission of the People’s Republic of China, 2016) was used to collect adult mosquitoes. Light traps are Kung Fu Xiaoshuai miniature (Photocatalytic Miewen Ying supply device; Wavelength: 2537 A˚; Power: 8 W; Corporation: Wuhan Environmental Protection Technology Co., Ltd. Gemstar), and they were hung at a height of 1.5 m above the ground. The research was conducted from April to November in 2018-2020. Select a fixed day for surveillance in a month for the sever prefectures (postponed in case of rain) and the investigation time was from 18:00 to the next morning. It consisted of two urban residential communities, two urban parks, two rural residential areas, and two rural livestock sheds with one light trap placed in non-privately owned areas of sampling sites in every surveillance city.

**Figure 1:**
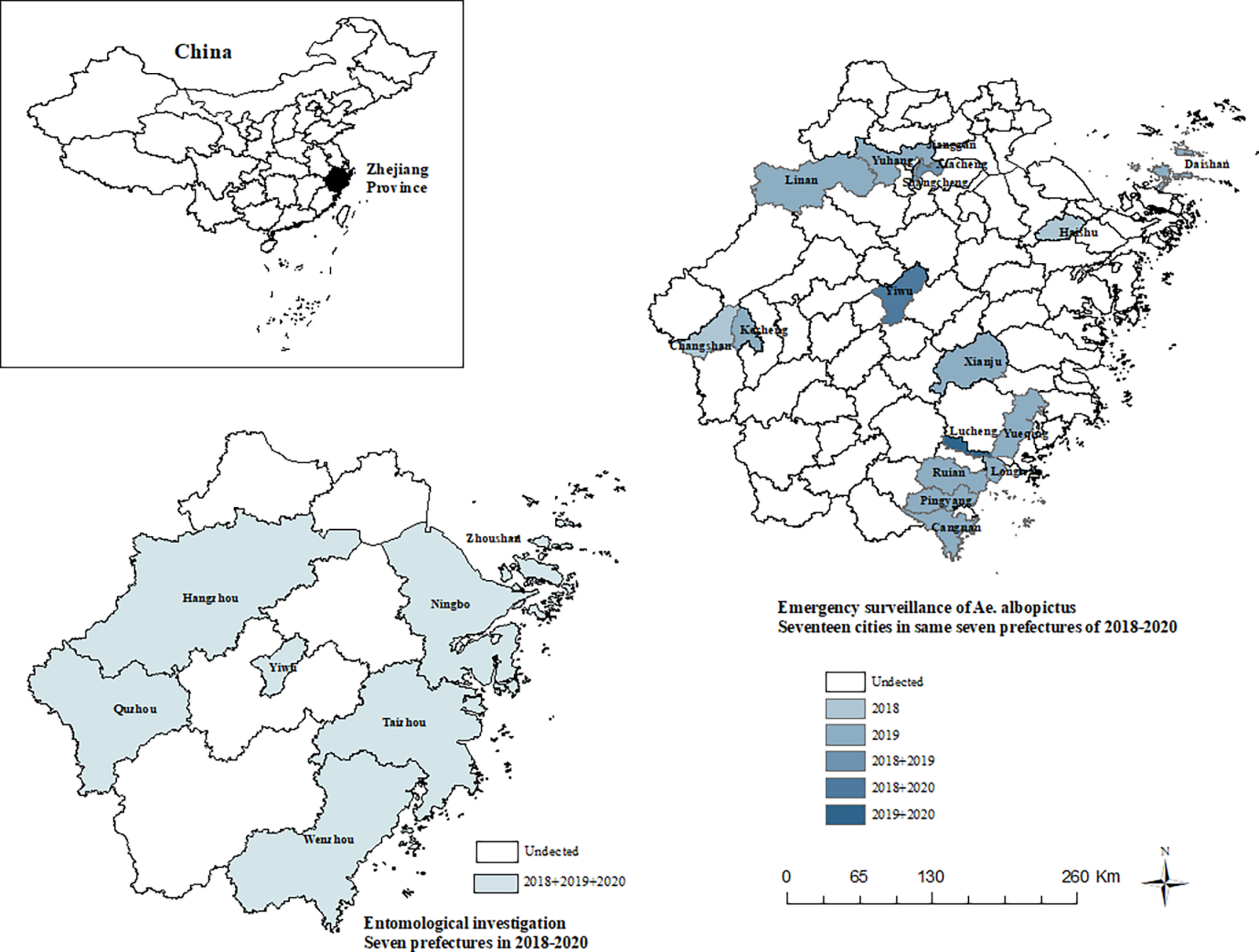
Study area for entomological investigation and emergency surveillance of *Ae. albopictus*.

The number of surveillance cities included in the seven prefectures is as follows: Taizhou (9), Yiwu (1), Hangzhou (15), Quzhou (6), Ningbo (10), Zhoushan (5), and Wenzhou (11) (**Figure 1**). Densities were calculated according to different mosquito species in different habitats. Mosquitoes were sexed and identified to species using “Fauna Sinica, Insecta, Eighth Volume, Diptera, Culicidae” (Lu, 1997a). Mosquito density (female adult/light trap/night) = number of female mosquitoes captured/[number of light traps * number of trapping nights]. Repeated one-way analysis of variance (ANOVA) was used to test the difference of “mean ± standard error” density of female mosquitoes of different species in different habitats in all entomological investigation areas. Pairwise differences in mosquito abundance were determined using the ANOVA Tukey-Kramer HSD *post-hoc* test with a significance level of 0.05 (p<0.05). Statistical analysis was carried out using SPSS 23.

### Emergency Surveillance of *Ae. albopictus*

Mosquitoes were collected in seventeen cities in Zhejiang province, China (**Figure 1**). Field collections were undertaken from summer to autumn from 2018 to 2020. The trapping period was 7:00-9:00 and 16:00-18:00, and the time of each collection was about 30min. Samples were collected in neighborhoods that previously or recently submitted notifications of cases of DF infections and traps were not placed in residents’ homes. Biogents Mosquitaire CO_2_ (Degener et al., 2019) (BG-Mosquitaire CO_2_) traps were placed on the sides of houses within a 200-m radius of a DF patient.

Mosquitoes were transported on dry ice to the laboratory and killed by freezing. Only *Ae. albopictus* were collected, and it was sexed and identified to species using “Fauna Sinica, Insecta, Eighth Volume, Diptera, Culicidae” (Lu, 1997a). The pools were formed according to male and female, year and region, and 2 ~ 35 per sample tube. The males were analyzed for transovarial transmission. Sampling tubes containing *Ae. albopictus* were placed on dry ice until they could be shipped to the Zhejiang Provincial Center for Disease Control and Prevention laboratory, where they were stored at -80°C until testing for viral RNA.

### Detection of Viral Genetic Material

To detect the presence of DENV, *Ae. albopictus* pools collected by emergency surveillance were prepared with 1 mL of the diluent containing Dulbecco’s modified Eagle’s medium (DMEM) (Gibco, Invitrogen) with 2% fetal bovine serum (FBS). Samples were homogenized with a Precellys Evolution (Bertin) homogenizer at a 45 Hz frequency for 1 min using 3 mm stainless steel beads and centrifuged for 10 minutes at 10000 rpm. A volume of 200 μL of the supernatant from each pool was used for RNA viral extraction with a QIAamp Viral RNA Mini Kit (Qiagen, Hilden, Germany) according to the manufacturer’s instructions.

Obtained RNA was subjected to RT–qPCR using a Dengue Fever Virus General-type Real Time RT–PCR Kit (Liferiver, China). If the sample was positive, a Dengue Fever Virus Genotyping I/II/III/IV Real Time RT–PCR Kit (Liferiver, China) was used to detect the type. The reaction was performed in an Applied Biosystems^®^ QuantStudio^®^ 5 instrument (Thermo Fisher, America), and cycle threshold values below 38 were considered positive.

The full-length genome of DENV-1 was sequenced directly from mosquito pool macerates. Sequences were obtained directly from the original samples; virus isolation was not attempted.

### Nucleotide Sequencing

Primers designed for the DENV-1 whole-gene sequence (Ma et al., 2016) were used for fragment amplification. The reaction had 12.5μLof 2×One Step Mix (Dye Plus), 4.25μL nuclease-free water, 1μL of forward and reverse primers respectively, 1.25μL One Step Enzyme Mix and 5μL of extracted RNA (HiScript^®^II One-step RT-PCR Kit (Dye Plus), Vazyme, China). Reverse transcription-polymerase chain reaction (RT-PCR) was carried out in one step with the following protocol: initial reverse transcription at 50°Cfor 30min; denaturation at 95°C for 5min, followed by 35 cycles 94°C for the 30s, 55°C for 30s, 72°C for 1min; and a final extension step at 72°Cfor 5min. Then the PCR products were analyzed *via* 1.2% agarose gel electrophoresis ultraviolet imaging. Finally, the resulting PCR products were purified and sequenced by the Sanger method using a Big Dye Terminator Sequencing Kit (ABI) in an ABI 3730XL Automated DNA Sequencer (ABI). The double-stranded DNA fragments were sequenced in both directions to generate a consensus double-stranded sequence for each sample. Multiple sequence alignment was performed using Bioedit Sequence Alignment Editor version 7.2.1 and trimmed to generate a dataset of 10,179 nucleotides (3,393 amino acids) encoding the complete DENV-1 open reading frame (ORF) and 1485 nucleotides (495 amino acids) encoding the E gene. The aligned sequences were used to analyze amino acid sequence variation and visualized in MeAlign of DNAStar.

All nucleotide sequences were aligned using ClustalW, and sequence polymorphisms within the amplified region were identified by comparing our sequences with a variety of sequences of the four dengue virus serotypes available in GenBank. A similarity tree was built with the maximum-likelihood based on a Tajima-Nei model, and nucleotide distances were determined by MEGA-X software (Molecular Evolutionary Genetics Analysis, version 10.0), with 1000 bootstrap replications. The names of each sequence in the similarity tree are displayed in the order of the GenBank number, separation place, and separation time.

## Results

### Entomological Investigation

On the whole, a total of 141607 female mosquitoes were caught through light trap method during 2018-2020. The proportions of different mosquitoes were *Culex pipiens quinquefasciatus/pallens* (*Cx. pipiens s.l*) (41.32%), *Culex. tritaeniorhynchus* (47.6%), *Ae. albopictus* (2.5%), *Anophelessinensis* (5.87%), *Armigeres subalbatus* (2.64%) and other mosquito species (0.07%) (**Supplementary Table 1**).

**Figure 2:**
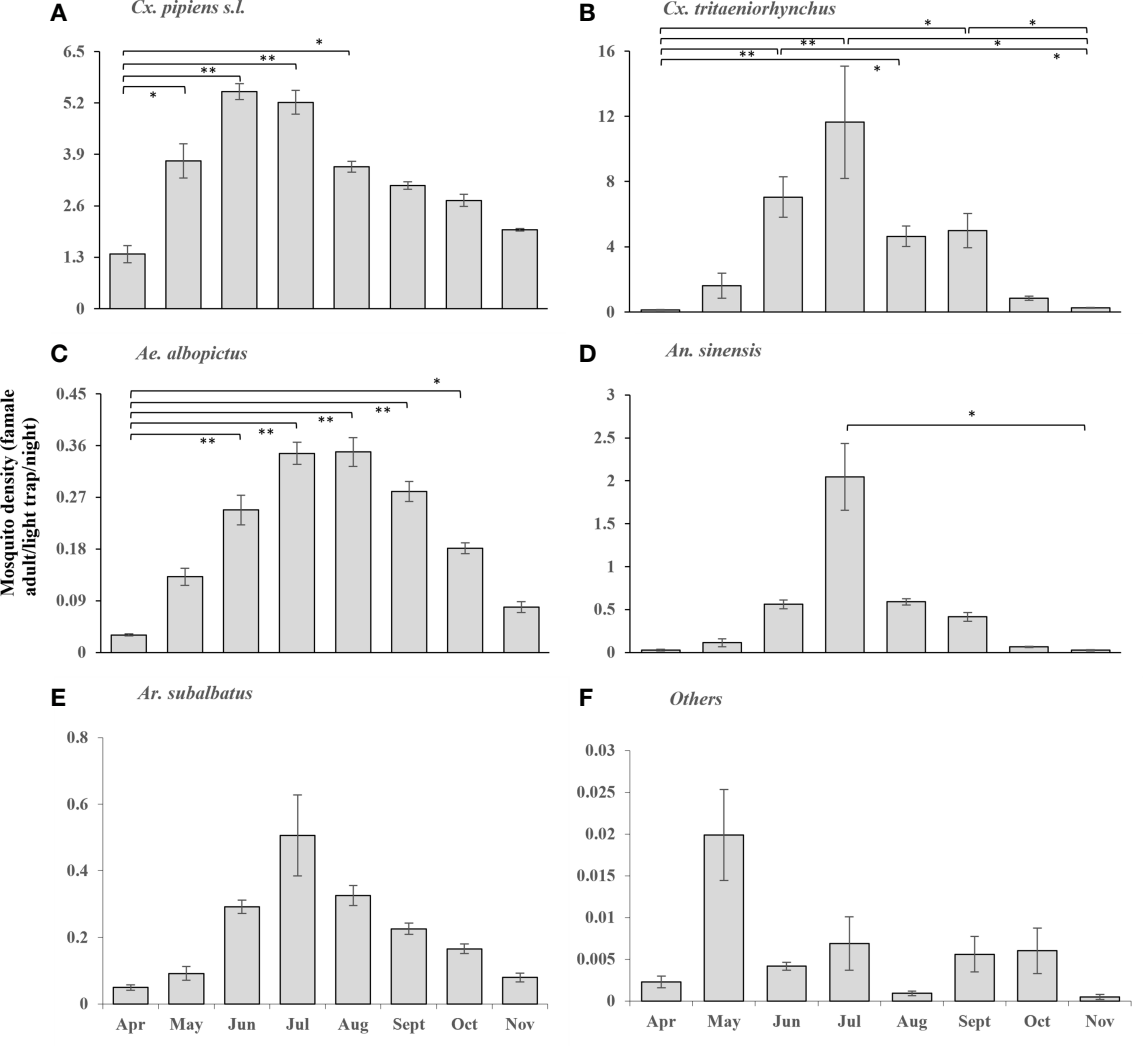
According to the entomological investigation, the densities of female mosquitoes of different species of different habitats in Zhejiang Province from April to November, in 2018-2020. **(A)** Cx. pipiens s.l.; **(B)** Cx. tritaeniorhynchus; **(C)** Ae. albopictus; **(D)** An. sinensis; **(E)** Ar. subalbatus; **(F)** Others. Values are the mean ± standard error. Different patterns indicate significantly different (*: p<0.05; **: p<0.01).

**Figure 3:**
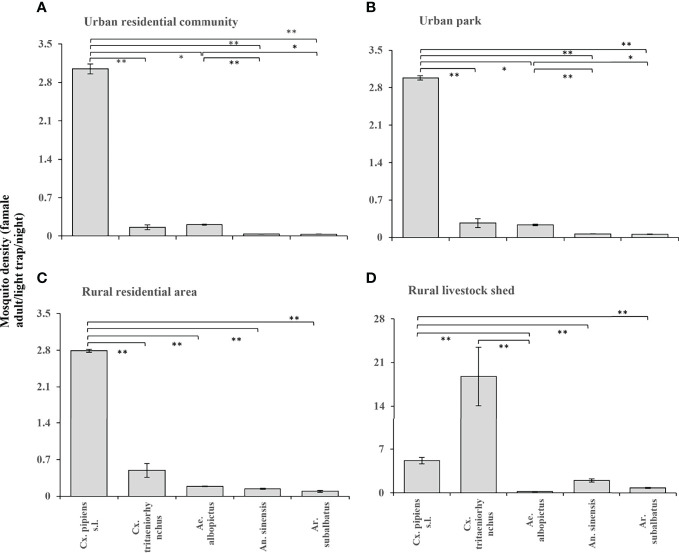
According to the entomological investigation from 2018 to 2020, the density of female mosquitoes of different species in different habitats. **(A)** Urban residential community; **(B)** Urban park; **(C)** Rural residential area; **(D)** Rural livestock shed. Values are the mean + standard error. Different patterns indicate significantly different (*: p<0.05; **: p<0.01).

From April 2018 to November 2020, it is showed that the density of female mosquitoes of different species in different habitats of seven prefectures in Zhejiang province (**Figures 2**, **3**). The annual surveillance started in April and ended in November, and basically showed an upward and then downward trend. *Cx. pipiens s.l* fluctuated at a high level from May to October, and the annual peak of *Cx. tritaeniorhynchus* was in July; in addition, the densities of the two mosquitoes of June and July was significantly higher than that in April (**Figures 2A, B**). The density of *Ae. albopictus* was low throughout the year, which generally began to appear in April, and there was a significant difference compared with the densities from June to September, respectively (**Figure 2C**). The peaks of *An. sinensis* and *Ar. subalbatus* were in July (**Figures 2D, E**). For other species of mosquitoes, there was no obvious dynamic trend throughout the year (**Figure 2F**).

*Cx. pipiens s.l.* were the dominant species in urban residential communities, urban parks, and rural residential areas and its density was significantly different from the other four mosquitoes (**Figures 3A–C**); in addition, the density of *Cx. tritaeniorhynchus* in rural residential areas was higher than that in the two urban habitats respectively. *Cx. tritaeniorhynchus* was the main dominant species in the rural livestock sheds, and its density was significantly different from that of *Ae. albopictus* (**Figure 3D**); while the density of *An. sinensis* and *Ar. subalbatus* was also higher than that of the other three habitats.

### Emergency Surveillance of *Ae. albopictus*

A total of 693 *Ae. albopictus* (664 females and 29 males) were collected and formed 49 pools. In 2018-2020, 73 female mosquitoes, 440 female and 6 male mosquitoes, 151 female and 23 male mosquitoes were collected, respectively. The characteristics of collected mosquitoes in each city are shown in **Supplementary Table 2**.

There were three circumstances of mosquito collection: The first circumstance: the sampling time before mosquito control during the local outbreak of DF in Lucheng of Wenzhou, 2019; The second circumstance: the sampling time after mosquito control during the local outbreak of DF of other cities in 2018-2019; The third circumstance: Past DF epidemic areas, where the sites without mosquito control in Lucheng of Wenzhou and Yiwu in 2020. The pools formed by mosquitoes collected in these three circumstances were 3 (6.1%), 35 (71.5%), and 11 (22.4%) respectively (**Table 1**).

**Table 1:** The three circumstances of *Ae. albopictus* collection in Zhejiang province during the 2018–2020 dengue outbreak.

The circumstance of mosquito collection	Females	Males	Number of pools tested	Positive pools
A local outbreak of DF, the sampling time before mosquito control (Lucheng of Wenzhou, 2019)	42	0	3	1
A local outbreak of DF, the sampling time after mosquito control (other cities, 2018-2019)	635	6	35	0
Past DF epidemic areas, the sites without mosquito control (Lucheng and Yiwu, 2020)	151	23	11	0
Total	664	29	49	1

Of the 49 pools tested, only one in the first circumstance was positive. The positive pool was composed of about fourteen female mosquitoes collected from the construction site (underground garage of a community) where DF cases were working in Lucheng District, Wenzhou in 2019. The pools composed of male mosquitoes were all negative, and the vertical transmission of DENV in *Ae. albopictus* was not detected in this study.

### Genome-Wide Phylogenetic Analysis

After whole-genome sequencing and splicing, the total length of the virus was 10,738 bp. The contents of the four bases were as follows: A (32.06%), T (21.46%), G (25.78%), C (20.70%), and G+C (46.48%). The lengths of the 5’ and 3’-untranslated regions were 94 and 465 nt, respectively. The only ORF was predicted to encode a large polyprotein of 3,392 amino acids. The complete genome sequence of DENV-1 ZJWZ2019 is available in GenBank under accession number OK448162.

### Whole-Genome Sequence Nucleotide and Amino Acid Homology Analysis

In the online basic local alignment search tool (BLAST) analysis, the nucleotide and amino acid homologies between ZJWZ2019 and Wenzhou DF serum isolate ZJWZ-62/2019 (MW582816) and ZJWZ-18/2019 (MW582815) were 99.96% and 99.92% and 99.97% and 99.91%, respectively. The nucleotide and amino acid sequences of ZJWZ2019 shared 99.74% and 99.91% homology with the 2019 strain from Cambodia (OK159963).

### Amino Acid Substitutions in the Whole Gene Sequence

Comparison of the genome of ZJWZ2019 with three closely related sequences showed that the variation among strains was very low. The protein regions of C, PrM, NS1, NS2A, NS4A, NS4B, and NS5 were highly conserved, while the amino acid changes were clustered in the E, NS2B, and NS3 protein regions. There were only five amino acid substitutions (**Table 2**).

**Table 2:** Comparison of amino acid differences in proteins in 4 DENV-1 strains.

Protein	Position	Cambodia (OK159963)	ZJWZ-18 (MW582815)	ZJWZ-62 (MW582816)	ZJWZ2019 (OK448162)
C (1-114)	–	–	–	–	–
PrM (115-280)	–	–	–	–	–
E (281-775)	437	E	E	E	K
	734	T	I	T	T
NS1 (776-1127)	–	–	–	–	–
NS2A (1128-1345)	–	–	–	–	–
NS2B (1346-1475)	1454	A	V	V	V
NS3 (1476-2094)	1696	I	V	I	I
	1824	Y	H	H	H
NS4A (2095-2244)	–	–	–	–	–
NS4B (2245-2493)	–	–	–	–	–
NS5 (2494-3392)	–	–	–	–	–

### Homology Analysis of the E Gene

In the online BLAST analysis, the nucleotide and amino acid homologies of the E gene between ZJWZ2019 and Wenzhou DF serum isolate ZJWZ-62/2019 were 99.87% and 99.80%, respectively. The E gene nucleotide homology with other Wenzhou DF serum isolates (ZJWZ-62/2019, ZJWZ-108/2019, ZJWZ-059/2019, ZJWZ-18/2019, ZJWZ-080/2019) was 99.8% ~ 99.9%, and the amino acid homology was 99.6% ~ 99.8%. The nucleotide homology with other domestic epidemic strains was 99.4~99.5%, and the amino acid homology was 99.6~99.8% (**Table 3**). Compared with the DENV-1 standard strain (AF425619), the amino acid sequence of the E protein of ZJWZ2019 has two glycosylation sites: position 67-69 (NTT), which was the same between the two; and position 153-155 (NET), which was changed to 153-155 (NES). The E Protein-related virulence sites E44 (E), E156 (T), and E366 (N) did not differ.

**Table 3:** Comparison of nucleotide and amino acid homologies of the E gene between ZJWZ2019 and the reference strain (%).

Number*	Strain name	1	2	3	4	5	6	7	8	9	10	11	12
1	ZJWZ2019	–	99.8	99.8	99.8	99.6	99.6	99.8	99.6	99.8	99.8	99.6	99.6
2	MW582816/ZJWZ-62/2019	99.9	–	100.0	100.0	99.8	99.8	100.0	99.8	100.0	100.0	99.8	99.8
3	MW582704/ZJWZ-108/2019	99.9	100.0	–	100.0	99.8	99.8	100.0	99.8	100.0	100.0	99.8	99.8
4	MW582702/ZJWZ-059/2019	99.9	100.0	100.0	–	99.8	99.8	100.0	99.8	100.0	100.0	99.8	99.8
5	MW582815/ZJWZ-18/2019	99.8	99.9	99.9	99.9	–	99.6	99.8	99.6	99.8	99.8	99.6	99.6
6	MW582703/ZJWZ-080/2019	99.8	99.9	99.9	99.9	99.9	–	99.8	99.6	99.8	99.8	99.6	99.6
7	OK159963/Cambodia/2019	99.5	99.7	99.7	99.7	99.6	99.6	–	99.8	100.0	100.0	99.8	99.8
8	OK159976/Cambodia/2019	99.5	99.6	99.6	99.6	99.5	99.5	99.8	–	99.8	99.8	99.6	99.6
9	MW228041/Cambodia/2019	99.5	99.6	99.6	99.6	99.5	99.5	99.8	99.9	–	100.0	99.8	99.8
10	MT705017/China/YN/2019	99.5	99.6	99.6	99.6	99.5	99.5	99.8	99.9	100.0	–	99.8	99.8
11	OK159958/Cambodia/2019	99.4	99.5	99.5	99.5	99.5	99.5	99.7	99.8	99.8	99.8	–	100.0
12	OK159937/Cambodia/2019	99.4	99.5	99.5	99.5	99.5	99.5	99.7	99.8	99.8	99.8	100.0	–

*The value in the lower left triangle is the percentage of amino acid homology (%), and the value in the upper-right triangle is the percentage of amino acid sequence homology (%).

### Phylogenetic Tree Analysis of Whole Gene Sequences

**Figure 4:**
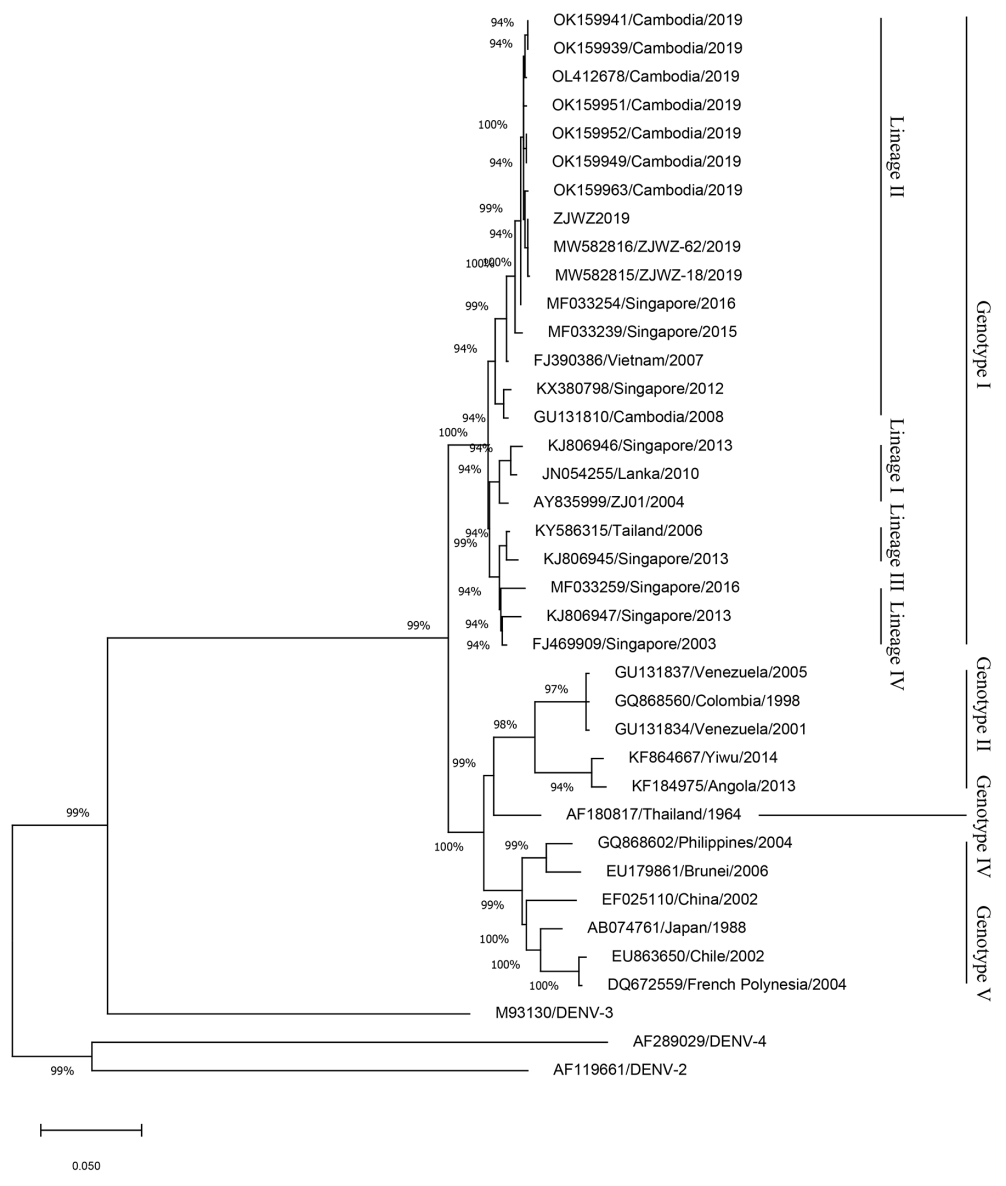
Phylogenetic tree based on the complete genome sequences of DENV-1.

The complete genome sequences of ZJWZ2019 and 37 sequences from representatives of different countries were chosen to construct a phylogenetic tree, and sequences of DENV-2, DENV-3, and DENV-4 were used as outgroups (**Figure 4**). The phylogenetic reconstruction classified all the DENV-1 isolates into four genotypes (genotype I, genotype II, genotype IV, genotype V), and four lineages (I-IV) of genotype I, and ZJWZ2019 was identified as genotype I, lineage II. ZJWZ2019 was closely related to ZJWZ-62/2019 and ZJWZ-18/2019 in Wenzhou, and they added OK159963/Cambodia/2019 as a secondary branch independently. Meanwhile, the other six isolates from Cambodia (OK159939/Cambodia/2019, OK159941/Cambodia/2019, OL412678/Cambodia/2019, OK159951/Cambodia/2019, OK159949/Cambodia/2019, OK159952/Cambodia/2019) are also on the same large primary branch as ZJWZ2019.

### Phylogenetic Analysis of DENV-1 Sequences Based on the E Gene

**Figure 5:**
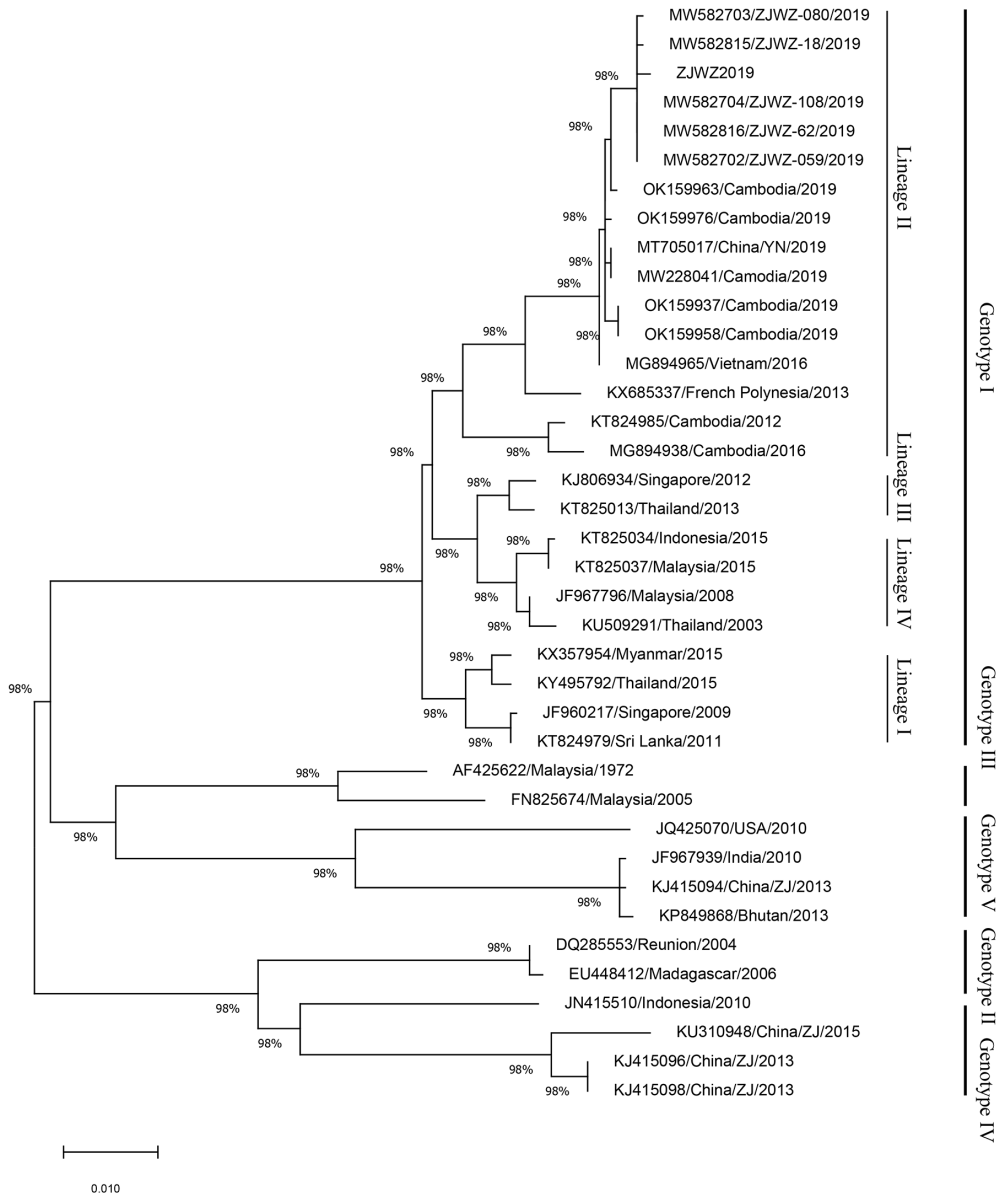
Phylogenetic analysis of DENV-1 sequences based on the E gene.

To further clarify the origin of ZJWZ2019, the E-gene sequences of ZJWZ2019 (E-ZJWZ2019) and 37 representative sequences from different countries were chosen to construct a phylogenetic tree (**Figure 5**). The phylogenetic reconstruction classified all the DENV-1 isolates into five genotypes (genotype I, genotype II, genotype III, genotype IV, genotype V), and four lineages (I-IV) of genotype I, and E-ZJWZ2019 was identified as genotype I, lineage II. E-ZJWZ2019 was closely related to five isolates (ZJWZ-62/2019, ZJWZ-108/2019, ZJWZ-059/2019, ZJWZ-18/2019, ZJWZ-080/2019) in Wenzhou, and OK159963/2019 in Cambodia formed a secondary branch independently.

## Discussion

Based on the entomological investigation, it was shown that the species composition, seasonal dynamics of mosquitoes in different habitats in Zhejiang province. *Cx. pipiens s.l.* accounted for a large proportion in urban residential communities, urban parks, and rural residential areas, which are house mosquito species and prefer the habitats related to the living environment of the human being. The density of *Cx. tritaeniorhynchus*, *An. sinensis* and *Ar. subalbatus* in rural habitats is higher than that in urban habitats. *Cx. tritaeniorhynchus* and *An. sinensis* mainly breed in rice fields, while rice is generally cultivated in rural environments, *Ar. subalbatus* breed in cesspits, which is common in rural areas. At the same time, the three mosquitoes prefer livestock blood, so they accounted for a relatively high proportion in two rural habitats (Lu, 1997a; Lu, 1997b).It is likely less influence of integrated management of *Ae. albopictus* during outbreak of DF on other species mosquito, Because the approach including of space spray on daytime and source reduction of container water, aim at the activities and breeding behaver *of Ae. albopictus*, the activity time of *Cx. pipiens s.l.*, *Cx. tritaeniorhynchus*, *An. sinensis* and *Ar. subalbatus* are at night, and containers are not main breeding sites for them (Lu, 1999).

The monitoring time of the light trap method was at night, not during the peak period of *Ae. albopictus* activity, so the capture rate of *Ae. albopictus* was very low. Therefore, when the DF occurs, it needs the application of other methods for surveillance of *Ae. albopictus*, such as BG traps for emergency (Roiz et al., 2016; National Health Commission of the People’s Republic of China, 2020).

In 2019, 22,599 cases of DF were reported in 28 provinces (municipalities, autonomous regions) in mainland China, and local outbreaks occurred in 13 provinces (municipalities, autonomous regions). That year was the year when the local epidemic of DF spread the farthest (Liu, 2020). Contemporaneously, a local epidemic of DF occurred in Wenzhou, Zhejiang (Yao et al., 2021), with a total of 290 cases reported. This was the first local outbreak since the first imported case was reported in 2004 (Yu et al., 2014). There were 93 imported cases, including 10 cases domestic and 83 cases overseas. The imported cases were mainly from Southeast Asian countries, of which approximately 70% were from Cambodia. A total of 197 indigenous cases were mainly concentrated in three districts (counties), including Lucheng District, where the positive mosquito samples were collected. The first indigenous case occurred in early August, the last case occurred in mid-October, and the onset time was concentrated in September. The information was collected from China’s disease prevention and control information system.

In early September 2019, DF occurred among construction workers in an underground garage in a newly built community in Lucheng District, Wenzhou, Zhejiang. Samples of mosquitoes were collected before the mosquito control program had been implemented, and DENV-1 strain ZJWZ2019 was detected in *Ae. albopictus*. ZJWZ-62/2019 and ZJWZ-18/2019 are the complete sequences of DENV-1 that have been entered into the NCBI library. It is indicated in the NCBI notes that both were isolated from the serum of Wenzhou DF cases on August 30, 2019. ZJWZ2019 compared with ZJWZ-62/2019 and ZJWZ-18/2019, whole sequence analysis showed 99.96% and 99.92% homology respectively, and E gene analysis showed 99.87% and 99.80% homology, respectively. It was confirmed that the sources of ZJWZ2019, ZJWZ-62/2019, and ZJWZ-18/2019 were consistent, which implies a natural transmission cycle of the dengue virus among humans and *Ae. albopictus*.

Similar to other RNA viruses, DENV is prone to variation due to the lack of an accurate correction mechanism during replication. Mutated viruses often cause regional DF epidemics. DENV-1 can be divided into five genotypes, with a 6% difference (Rico-Hesse, 1990; Fahri et al., 2013). The whole-genome phylogenetic tree shows that the phylogenetic relationship between ZJWZ2019 and ZJWZ-62/2019 and ZJWZ-18/2019 is the closest. The evolutionary relationship between ZJWZ2019 and the Cambodia virus strain (OK159963) is similar, suggesting that ZJWZ2019 may have come from Cambodia. OK159963, according to NCBI notes, was derived from the serum of a DF patient in Cambodia in July 2019. ZJWZ2019, OK159963, along with some Cambodia strains of 2019 and other Southeast Asian strains, these strains formed a large cluster belonging to lineage II. There were cases imported into Wenzhou from Cambodia in 2019, which suggests the possibility of local outbreaks in the Lucheng district caused by imported cases from the region.

The results of this study present a relatively complete possible transmission chain for the local DF outbreak in the Lucheng district of Wenzhou, Zhejiang, in 2019. Dengue virus imported from Cambodia infected local *Ae. albopictus*, and after the external incubation period, *Ae. albopictus* transmitted DENV-1 to residents, resulting in the outbreak of local DF. The evolutionary tree of the E gene also verified this transmission chain.

The E protein is the main structural protein of DENV, which plays an important role in virus infection, immunity, and immune injury (Modis et al., 2004). E155 in the E protein is a glycosylation site. If this site is changed, it will affect the virulence of the virus, which has been confirmed in DENV-1 (Puri et al., 1997). This study found that E155 in ZJWZ2019 changed (T-S), and whether changes in other amino acid sites on DENV-1 will cause changes in virus cell tropism and virulence needs to be further studied.

There is only a small likelihood of isolating DENV from *Aedes* mosquitoes. Even during the DF outbreak in Guangdong in 2014, a large number of DENV virus strains were isolated from human serum samples, but there were few reports of the detection or isolation of DENV virus from vectors (Sun et al., 2016). The reasons may be related to the collection time and place, the preservation and transportation of samples, the timely detection and isolation of the virus, and low infection rates in mosquito populations. In this study, the mosquito collection time was in September, corresponding to the most concentrated time of the DF outbreak in Wenzhou. After the collection of *Ae. albopictus*, the samples were preserved on dry ice, transported to the laboratory and stored in a -80 °C freezer. Finally, a dengue virus strain was successfully detected. In addition to the above reasons, it may also be due to the lag in the sampling time. Most mosquito samples were obtained after mosquito control treatment at the dengue epidemic location.

Traditional epidemiological investigations have difficulty tracing sources of local outbreaks. The whole gene sequence of the virus can be obtained by detecting the pathogen carried by vector mosquitoes and isolating the virus strain, which can be used for the analysis and research of virus gene evolution. By understanding the genetic characteristics of the virus, the source of DF can be traced more accurately. In this sense, whole gene sequencing and phylogenetic analysis are important tools to study the dynamics of infection, with major impacts on surveillance and vector control programs that aim to prevent dengue outbreaks.

Some limitations must be acknowledged in the entomological investigation. Firstly, when the light trap method was used for surveillance, only five common mosquitoes were identified, it needs more habits of mosquito selected as surveillance sites, such as forest and mountain area. Secondly, when using BG-Mosquitaire CO_2_ for surveillance, the monitoring time was short, and only a small number of *Ae. albopictus* were collected; because the requirements of vector *Aedes* surveillance guidelines need to be taken into account.

In conclusion, a total of 141607 female mosquitoes were caught through the light trap method. There were five common species in Zhejiang Province: *Cx. pipiens s.l*, *Cx. tritaeniorhynchus*, *Ae. albopictus*, *An. sinensis* and *Ar. subalbatus*. In the emergency surveillance and pathogen detection of *Ae. albopictus*, a total of 693 were collected and 49 pools were formed, only one pool was positive. By sequencing the positive sample, the full-length sequence of a DENV-1 virus strain was obtained and identified by molecular biological analysis. It was traced that the virus strain causing the local outbreak of DF in Lucheng District of Wenzhou in 2019 may come from Cambodia.

## Data Availability Statement

The datasets presented in this study can be found in online repositories. The names of the repository/repositories and accession number(s) can be found below: https://www.ncbi.nlm.nih.gov/, OK448162.

## Author Contributions

TZ and ZG conceived the study and coordinated its implementation. ZG, TZ, and QL participated in the experimental design. QL, JW, JH, and YW collected mosquito samples. ZG and QL performed the experiments. YJ, XG, CL, JG, HZ, DX, and QL interpreted the data. QL and TZ drafted the manuscript. All authors contributed to the article and approved the submitted version.

## Funding

This work was funded by grants from the Infective Diseases Prevention and Cure Project of China (no. 2017ZX10303404).

## Conflict of Interest

The authors declare that the research was conducted in the absence of any commercial or financial relationships that could be construed as a potential conflict of interest.

## Publisher’s Note

All claims expressed in this article are solely those of the authors and do not necessarily represent those of their affiliated organizations, or those of the publisher, the editors and the reviewers. Any product that may be evaluated in this article, or claim that may be made by its manufacturer, is not guaranteed or endorsed by the publisher.
